# The common and distinct neural bases of affect labeling and reappraisal in healthy adults

**DOI:** 10.3389/fpsyg.2014.00221

**Published:** 2014-03-24

**Authors:** Lisa J. Burklund, J. David Creswell, Michael R. Irwin, Matthew D. Lieberman

**Affiliations:** ^1^Department of Psychology, University of CaliforniaLos Angeles, Los Angeles, CA, USA; ^2^Department of Psychology and Center for the Neural Basis of Cognition, Carnegie Mellon UniversityPittsburgh, PA, USA; ^3^Department of Psychiatry and Biobehavioral Sciences, Cousins Center for Psychoneuroimmunology, University of CaliforniaLos Angeles, Los Angeles, CA, USA; ^4^Department of Psychiatry and Biobehavioral Sciences, University of CaliforniaLos Angeles, Los Angeles, CA, USA

**Keywords:** affect labeling, reappraisal, emotion regulation, fMRI, amygdala, prefrontal cortex

## Abstract

Emotion regulation is commonly characterized as involving conscious and intentional attempts to change felt emotions, such as, for example, through reappraisal whereby one intentionally decreases the intensity of one's emotional response to a particular stimulus or situation by reinterpreting it in a less threatening way. However, there is growing evidence and appreciation that some types of emotion regulation are unintentional or incidental, meaning that affective modulation is a consequence but not an explicit goal. For example, affect labeling involves simply verbally labeling the emotional content of an external stimulus or one's own affective responses without an intentional goal of altering emotional responses, yet has been associated with reduced affective responses at the neural and experiential levels. Although both intentional and incidental emotional regulation strategies have been associated with diminished limbic responses and self-reported distress, little previous research has directly compared their underlying neural mechanisms. In this study, we examined the extent to which incidental and intentional emotion regulation, namely, affect labeling and reappraisal, produced common and divergent neural and self-report responses to aversive images relative to an observe-only control condition in a sample of healthy older adults (*N* = 39). Affect labeling and reappraisal produced common activations in several prefrontal regulatory regions, with affect labeling producing stronger responses in direct comparisons. Affect labeling and reappraisal were also associated with similar decreases in amygdala activity. Finally, affect labeling and reappraisal were associated with correlated reductions in self-reported distress. Together these results point to common neurocognitive mechanisms involved in affect labeling and reappraisal, supporting the idea that intentional and incidental emotion regulation may utilize overlapping neural processes.

## Introduction

Emotion regulation refers to processes that alter the character or intensity of emotional experiences. The capacity to effectively regulate negative emotional experiences, in particular, is essential for healthy mental and physical functioning (Gross and Thompson, 2007). Such control helps us navigate and survive the inevitable ups and downs of everyday life. While emotion regulation is commonly thought of as referring to conscious and intentional attempts to change felt emotions, there is growing evidence and appreciation that some types of emotion regulation are unintentional, automatic, or incidental byproducts of processes set in motion to serve non-regulatory goals (Mauss et al., 2007; Berkman and Lieberman, 2009; Berkman et al., 2009; Koole and Rothermund, 2011). As such, various emotion regulation strategies may be categorized as either “intentional” or “incidental.” While intentional and incidental emotion regulation seem to have similar emotion-modulatory effects, little research has directly compared their underlying neural mechanisms. Therefore, in this study, we compared intentional and incidental emotion processing, namely, reappraisal and affect labeling, at the neural and experiential levels, to examine the extent to which they involve common vs. distinct neural and psychological mechanisms.

Reappraisal is one of the most commonly used and studied emotion regulation strategies (Gross and Thompson, 2007; Kalisch, 2009; Hartley and Phelps, 2010). Reappraisal involves intentionally decreasing the intensity of one's emotional response to a particular stimulus or situation by reinterpreting it in a less threatening way. A person is using reappraisal when she tries to “look on the bright side” or “find the silver lining” of an undesirable event or situation. In fact, developing effective reappraisal skills is one of the core components of cognitive behavioral therapies for anxiety disorders, which ostensibly represent pathologically poor emotion regulation (Craske, 2003; Gross and Thompson, 2007). At the neural level, reappraisal has been associated with increased activity in ventrolateral prefrontal cortex (VLPFC), posterior dorsomedial prefrontal cortex (DMPFC), and dorsolateral prefrontal cortex (DLPFC), although the precise location of activations in these areas have varied across studies, perhaps due to differences in specific stimuli, response timing, and task instructions (Berkman and Lieberman, 2009; Kalisch, 2009; Ochsner and Gross, 2005). Reappraisal is also consistently associated with consequential decreases in amygdala activity and self-reports of distress (see Ochsner and Gross, 2005; Quirk and Beer, 2006; Phillips et al., 2008; Berkman and Lieberman, 2009 for reviews).

Affect labeling typically involves verbally labeling the emotional content of a stimulus, such as labeling an angry facial expression as “angry” or a fearful facial expression as “fearful” (Hariri et al., 2000, 2003; Lieberman et al., 2005, 2007). Although such affect labeling may not seem like an emotion regulation strategy on the surface—mainly because it does not involve an intentional goal of changing felt emotions—several findings suggest that it constitutes a form of incidental emotion regulation. Like reappraisal, this form of affect labeling while viewing aversive images is associated with diminished self-reports of distress (Lieberman et al., 2011), and in translational studies, has produced physiological or behavioral outcomes associated with a reduced fear response (Tabibnia et al., 2008). Additionally, several studies have demonstrated a pattern of neural responses during this type of affect labeling that overlaps with the pattern for reappraisal, primarily including increased VLPFC activity and decreased amygdala activity (Hariri et al., 2000, 2003; Lieberman et al., 2005, 2007; Foland et al., 2008; Payer et al., 2011; Gee et al., 2012).

In the present study, we examined a more personally-relevant form of affect labeling. Specifically, we modified the typical affect labeling instructions so that participants labeled their own emotional responses to aversive stimuli rather that simply labeling the emotional content of external stimuli. Accordingly, this form of affect labeling constitutes a more ecologically-valid form of incidental emotion regulation since affect labeling in everyday life and therapeutic contexts likely involves labeling one's own affective reactions. One previous study found that labeling one's own emotional responses resulted in reduced fear responding, including decreased physiological and behavioral outcomes (Kircanski et al., 2012; see also Satpute et al., 2012). These findings suggest that this form of affect labeling likely produces similar effects as labeling the affective content of external stimuli. However, no previous studies to our knowledge have examined the neural bases of labeling one's own emotional responses and the consequences for diminishing affective responses.

Despite apparent similarities in the consequences and mechanisms of affect labeling and reappraisal, only a few studies have compared them to identify specific functional similarities and differences. In one behavioral study, Lieberman et al. (2011) found that labeling external emotional stimuli and reappraisal produced correlated reductions in self-reported distress such that individuals who benefited from one strategy tended to benefit from the other as well. A second study (Payer et al., 2012) examined neural responses during reappraisal and affect labeling of external emotional stimuli measured on different days in a small sample (*N* = 9). Anatomical ROI analyses yielded correlated reductions in amygdala activity from the two emotion processing strategies such that those with larger reductions in amygdala activity during reappraisal also tended to have larger reductions in amygdala activity during affect labeling. No previous studies to our knowledge have compared the neural bases of reappraisal and personally-relevant affect labeling.

The current investigation examines both common and divergent neural and self-report effects of personally-relevant affect labeling and reappraisal. A large sample was included (*N* = 39) to avoid any issues of statistical power. We hypothesized that personally-relevant affect labeling and reappraisal would engage multiple overlapping lateral prefrontal regulatory regions, including VLPFC, and result in decreased amygdala responses and self-reported distress. We also hypothesized that reappraisal would result in more widespread prefrontal activation than affect labeling because it seems to represent a more complex effortful psychological process (Kalisch, 2009). Results of this study will enhance our understanding of how we can modulate our emotions, which has both basic and clinical implications. For example, identifying specific common neural regions in this study will lay the groundwork for future studies to explore the precise mechanisms that are most effective in regulating affective responses. Additionally, evidence verifying common underlying neural mechanisms for intentional and incidental regulation would support further research into novel treatment strategies for emotion-related disorders that focus on enhancing incidental emotion regulation which may be less aversive and thereby more appealing to certain patients, resulting in decreased rates of treatment dropout.

## Methods

### Participants

Participants (Ps) aged 55–85 years were recruited from the UCLA community and Los Angeles area via newspaper advertisements to participate in the study as part of a larger project examining the effects of an 8-week mindfulness-based stress reduction (MBSR) program. If deemed potentially eligible during a telephone screening, participants were scheduled to come to the UCLA campus to complete a detailed, in-person screening. Of 70 individuals invited to complete an in-person screening, 41 were eligible and participated in the study. Two Ps were excluded from the analyses due to excessive head motion during fMRI scanning, leaving a final sample for the fMRI results of 39 Ps ranging in age from 55 to 75 (*M* = 64.65, *SD* = 7.25; 8 male, 31 female). Self-report data collected during scanning, as described below, is missing for an additional two individuals due to technical difficulties and thus, self-report results reflect a sample of 37 people. All individuals provided informed consent prior to completing the in-person screening. Importantly, the MBSR intervention components of the study were all subsequent to the scanning reported on here. Results of the larger intervention study will be published in a separate manuscript.

#### Inclusion and exclusion criteria

Inclusion criteria were: (a) English-speaking adults 55–85 years of age at time of entry; (b) post-menopausal and not pregnant, if female; and (c) willing and able to come to UCLA for all study related activities. Participants were excluded if they: (a) were not ambulatory, (b) indicated any treatment for mental health problems in the last 6 months, (c) indicated any major physical health problems in the last 3 months, (d) used medications affecting cardiovascular or endocrine function, (e) indicated regular use of psychotropic medication or psychotherapy in the last 6 months, (f) indicated any use of doctor-prescribed cholesterol lowering medications (e.g., statins), (g) used any doctor-prescribed pain medication, (h) indicated any implants, (i) exhibited cognitive impairment as indicated by a score lower than 23 on the Mini-Mental State examination (Folstein et al., 1975), (j) smoked, (k) practiced regular (>1 time per week) mind-body therapy (e.g., meditation, yoga, tai chi) anytime in the last 6 months, (l) were left-handed, (m) had non-removable metal in their bodies (other than dental fillings), (n) indicated feeling claustrophobic in confined spaces, such as an fMRI scanner, and/or (o) weighed over 300 lbs^1^.

#### General procedures

Following the in-person screening, eligible, and interested participants were scheduled to complete an fMRI session, during which they completed the affect labeling and reappraisal task described below. Immediately prior to entering the scanner, participants were given detailed instructions on the different conditions of the task and given a chance to practice the task. Participants discussed each of their responses with the experimenter to make sure they understood the instructions and were able to adequately complete the task prior to being scanned. All scanning sessions were completed prior to participants beginning the MBSR intervention. Participants were paid $50 for their participation, plus $16 reimbursement for parking. Our research protocol was approved by the UCLA Office for the Protection of Human Research Subjects' Institutional Review Board.

#### fMRI affect labeling and reappraisal task

**Figure 1:**
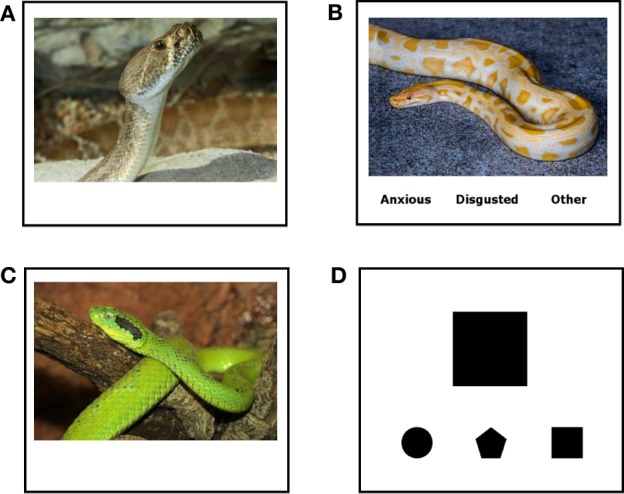
**Stimuli**. Sample screens from the **(A)** Reappraisal, **(B)** Label, **(C)** Observe, and **(D)** Shape Match conditions. Although specific images for Reappraisal, Label, and Observe were distinct, they were matched on content, valence, and arousal. (Note: The pictures shown in Figure 1 are similar to the stimuli used in this study, but are not actual IAPS images.)

While being scanned, participants were shown photographs of negative emotionally-evocative scenes (from the International Affective Picture System; IAPS; Lang et al., 2008, as described below) presented in a blocked design and were instructed to either passively view the stimuli, or use one of two emotion regulation strategies, affect labeling or reappraisal. A non-emotional shape-matching task was also included as a control condition indexing simple cognitive-motor responses. Other conditions, such as neutral and positive scenes were included, but are not discussed further in this manuscript. Thus, there were four conditions (i.e., types of trials) that were analyzed for this manuscript (see Figure 1). (1) In trials for the observe condition (Observe), participants passively viewed a single negative emotionally-evocative scene. For this condition, participants were told to simply look at each picture. (2) In trials for the affect label condition (Label), participants viewed a single negative emotionally-evocative scene and chose the label from three words at the bottom of the screen that best matched their own emotional response to the scene. For this condition, participants were presented with three response options taken from the following possibilities, “Sad,” “Anxious,” “Disgusted,” and “Other.” Importantly, “Other” was always offered as an option in case participants experienced an emotion not captured by the other response options or did not have any emotional response at all. The response options were balanced across blocks, with the order of the response options counterbalanced across blocks. (3) In trials for the reappraise condition (Reappraise), participants viewed a single negative emotionally-evocative scene and used cognitive reappraisal to decrease their emotional response to the scene. Specifically, participants were told to try to decrease their emotional response to each image by thinking about it in less distressing terms and were given examples of how to accomplish this. For instance, if they saw a picture of a woman in the hospital, rather than imaging how bad this is, they could think about how the woman was in the hospital to get better and would be healthy again very soon. They could also think that a distressing scene was not real but instead just a scene from a movie constructed using special effects. (4) In trials for the shape-matching condition (Shape Match), participants saw a target geometric shape and chose the shape from three options at the bottom of the screen that matched the top target shape. Blocks of trials were separated by a fixation crosshair. As mentioned above, participants were given ample opportunity to practice all conditions until they felt comfortable using each strategy prior to entering the scanner.

Negative scene stimuli were taken from the IAPS set (Lang et al., 2008). Negative scenes were selected as those with the highest combined negative valence and arousal, based on previous IAPS ratings (Lang et al.). These valence and arousal ratings were matched across the Observe, Label, and Reappraise conditions [valence: *F*_(2)_ = 0.004; *p* = 0.99; arousal: *F*_(2)_ = 0.046; *p* = 0.96]. The normative valence and arousal ratings for each condition were as follows: Observe-valence *M*(*SD*) = 2.79(1.05); Observe-arousal = 5.94(0.76); Label-valence = 2.80(0.95); Label-arousal = 5.96(0.65); Reappraise-valence = 2.82(0.86); Reappraise-arousal = 5.89(0.83). Additionally, the content of the scenes (e.g., snake, burn victim, funeral scene) was matched across these three conditions^2^. The specific stimulus-condition pairings were the same for all participants.

There were 4 blocks of each of the 4 condition types, with each block consisting of 5 trials. Each trial was 6 s long, with the stimulus presented for the entire trial length. Each block was preceded by a 10-s fixation crosshair, during which time participants were instructed to focus their attention on the crosshair, and a 5-s instruction cue indicating the condition type for that block (i.e., Observe, Affect Label, Reappraise, or Shape Match). Immediately following each block, participants provided ratings of how unpleasant they felt on average during the immediately preceding block, on a scale from 0 to 3 where 0 = “Not at all unpleasant,” 1 = “Somewhat Unpleasant,” 2 = “Moderately Unpleasant,” and 3 = “Severely Unpleasant.” Four random orders of the conditions were created and counterbalanced across participants.

Stimuli were presented on a Macintosh computer using MacStim 3.2.1 software (WhiteAnt Occasional Publishing, www.brainmapping.org/WhiteAnt) and high-resolution magnet-compatible goggles (Resonance Technology, Inc). Button press responses were collected using an MR-compatible button box connected to the Macintosh.

#### Image acquisition

Data were acquired on a Siemens Sonata 1.5T scanner at the UCLA Ahmanson-Lovelace Brainmapping Center. Head movements were restrained with foam padding. High-resolution structural T2-weighted echo-planar images (spin-echo; *TR* = 5000 ms; *TE* = 33 ms; matrix size 128 × 128; 32 axial slices; FOV = 20-cm; 3-mm thick, skip 1-mm) were acquired coplanar with the functional scans. Eight functional scans were acquired (echo planar T2^*^-weighted gradient-echo, *TR* = 3000 ms, *TE* = 25 ms, flip angle = 90°, matrix size 64 × 64, 32 axial slices, *FOV* = 20-cm; 3-mm thick, skip 1-mm). Voxel size = 3.125 × 3.125 × 3 mm.

#### fMRI data analysis

The imaging data were analyzed using Statistical Parametric Mapping (SPM5; Wellcome Department of Cognitive Neurology, Institute of Neurology, London, UK) and MarsBaR (Brett et al., 2002), a region of interest (ROI) toolbox. Functional images for each participant were realigned to correct for head motion, coregistered to the high-resolution structural images, normalized into a standard stereotactic space as defined by the Montreal Neurological Institute (MNI), and smoothed with an 8 mm Gaussian kernel, full width at half maximum, to increase signal-to-noise ratio. Final voxel size for all analyses was 3 × 3 × 3 mm. Experimental blocks were modeled using a boxcar function convolved with the canonical hemodynamic response. Linear contrasts were computed for each participant using a fixed-effects model. For group analyses, contrast images were pooled together in random-effects analyses, ensuring valid inference to the sampled population.

To examine regions commonly activated during affect labeling and reappraisal, we used SPM5 to assess the conjunction null hypothesis, which yields voxels that are significantly activated across multiple contrasts. Specifically, we assessed the conjunction null hypothesis for two sets of contrasts, first, Label > Observe and Reappraise > Observe, and second, Label < Observe and Reappraise < Observe. We examined common activity for affect labeling and reappraisal relative to passive observation of negative scenes specifically in order to identify activations reflecting the “regulatory” aspect of these conditions rather than merely the perception of negative emotionally-evocative stimuli. Specifically, comparison against the Observe condition controls for processes involved in the passive observation of negative stimuli. To examine regions differentially activated for affect labeling and reappraisal, we examined group-level main effects analyses for the contrasts directly comparing affect labeling and reappraisal (i.e., Label > Reappraise and Label < Reappraise).

Given our strong a priori hypotheses regarding activity in specific brain regions (i.e., bilateral amygdala and PFC), our analyses focused on these regions of interest (ROIs) which were defined using an anatomical atlas (Automatic Anatomical Labeling atlas, AAL; Tzourio-Mazoyer et al., 2002) within SPM5. Analyses were statistically thresholded using either an uncorrected conjunction *p*-value threshold of 0.003 (Price and Friston, 1997; conjunction analyses only) or a standard uncorrected *p*-value threshold of 0.005 (main effects analyses only), coupled with cluster-extent significance thresholds of 4 and 24 contiguous voxels for the amygdala and entire PFC, respectively, which accounts for multiple comparisons in each ROI as calculated using the program AlphaSim (http://afni.nimh.nih.gov/afni/doc/manual/AlphaSim). Activations in other brain regions were thresholded using the same *p*-value thresholds as described above, coupled with a cluster-extent significance threshold of 43 contiguous voxels, which accounts for multiple comparisons across the whole brain based on calculations using AlphaSim.

Finally, given that affect labeling and reappraisal utilized different response modalities (i.e., option selection with button press vs. free-form cognition), we repeated the analysis directly comparing Label > Reappraise, but additionally used a technique designed to isolate activity consistent with the motor response processes unique to affect labeling. We first created a whole-brain mask based on the results of a one-sample *t*-test for the contrast Shape Match vs. implicit baseline, consisting of a fixation crosshair, thresholded at *p* < 0.005. This mask ostensibly isolates activity corresponding to response selection with a corresponding motor button press, in the absence of any emotional processing. As such, when this mask is applied to the analysis comparing Label > Reappraise, any remaining significant activations may reflect differences in response modality since they overlap with the activations seen during simple motor response, whereas activations that are no longer significant when this mask is applied do not overlap with the regions involved in motor response selection and therefore likely reflect differences other than those underlying response modality.

## Results

### Self-reported data

**Figure 2:**
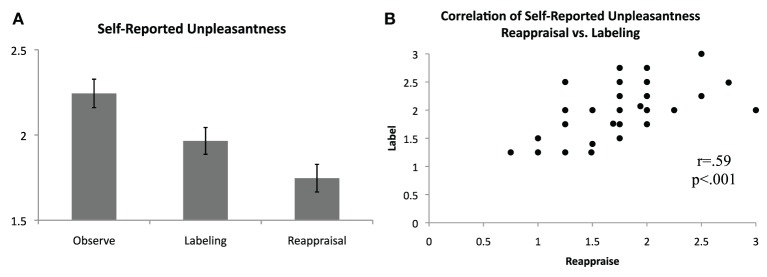
**Self-reported unpleasantness. (A)** Average self-reported unpleasantness, as rated immediately following blocks of passive observation, affect labeling, and reappraisal, on a scale from 0 (not at all unpleasant) to 3 (extremely unpleasant). **(B)** Significant positive correlation of self-reported unpleasantness for the reappraisal and affect labeling conditions.

Following each block, participants rated how unpleasant they felt during the preceding block, on average, using a scale of 0 (not at all unpleasant) to 3 (severely unpleasant). Consistent with predictions and as shown in Figure 2, unpleasantness ratings were significantly lower for the Label [*M*(*SD*) = 1.96(0.48)] and Reappraise [*M*(*SD*) = 1.75(0.49)] conditions relative to Observe [*M*(*SD*) = 2.24(0.51)], suggesting that both affect labeling and reappraisal were effective in reducing self-reported distress [Label vs. Observe: *t*_(36)_ = 3.59, *p* = 0.001; Reappraise vs. Observe: *t*_(36)_ = 5.74, *p* < 0.001]. Directly comparing the two regulation strategies, Reappraisal was associated with lower self-reported unpleasantness than Labeling [*t*_(36)_ = 3.02, *p* = 0.005]. The zero-order correlations for the three conditions were significant and positive [Label vs. Reappraise: *r* = 0.59, *p* < 0.001; Label vs. Observe: *r* = 0.54, *p* = 0.001; Reappraise vs. Observe: *r* = 0.44, *p* = 0.006], suggesting a similar response pattern in general for each participant across conditions. Nevertheless, self-reported unpleasantness ratings during Label and Reappraise continued to be significantly positively correlated even when controlling for ratings during Observe (*pr* = 0.46, *p* = 0.005), indicating that to the extent individuals were able to use reappraisal to reduce their self-reported unpleasantness, they were proportionately able to use affect labeling to reduce their self-reported unpleasantness.

### Neural responses common to affect labeling and reappraisal relative to observe

**Figure 3:**
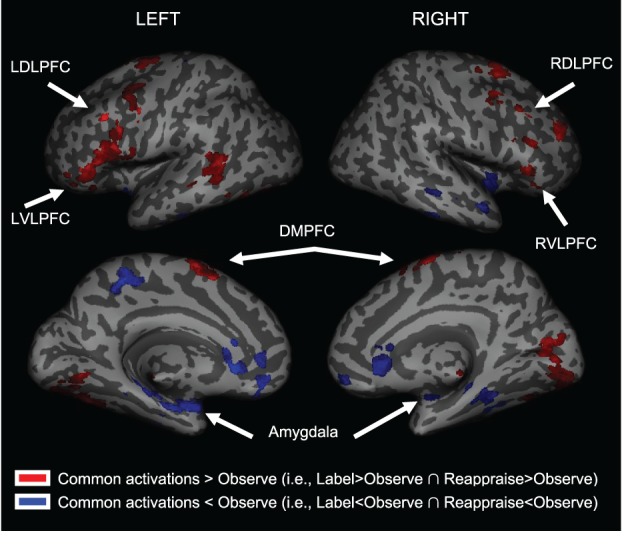
**Common activations for Labeling and Reappraisal relative to Observe only**. Areas in red were commonly activated relative to the Observe condition. Areas in blue were commonly deactivated relative to the Observe condition.

We first examined common *increased* activations for Label and Reappraise relative to Observe (i.e., the conjunction of Label > Observe and Reappraise > Observe). As shown in Table 1 and Figure 3, we found increased activations in RVLPFC (51, 27, 0; *t* = 3.23; 29 voxels), RDLPFC (51, 24, 45; *t* = 2.92; 106 voxels; and 21, 57, 30; *t* = 2.67; 65 voxels), a large cluster of activity on the left side (505 voxels) extending from LVLPFC (−54, 30, 6; *t* = 3.22) to LDLPFC (−33, 3, 63; *t* = 3.71), as well as posterior DMPFC (0, −6, 75; *t* = 3.90; 519 voxels; 12, 15, 48; *t* = 2.00; 27 voxels) during Label and Reappraisal relative to Observe. Together, these findings suggest that affect labeling and reappraisal utilize a number of overlapping prefrontal regions that have been associated with affect labeling and/or reappraisal in separate previous studies (Ochsner and Gross, 2005; Berkman and Lieberman, 2009; Kalisch, 2009).

**Table 1:** **Common activations for Labeling and Reappraisal relative to Observe-only**.

		***x***	***y***	***z***	***t***	**Voxels**
**COMMON REGIONS OF ACTIVATION (CONJUNCTION OF LABEL > OBSERVE AND REAPPRAISE > OBSERVE)**						
VLPFC/inferior frontal gyrus	R	51	27	0	3.23	29
VLPFC/inferior frontal gyrus	L	−54	30	6	3.22	505
DLPFC/middle frontal gyrus	L	−57	21	33	3.17	*
DLPFC/superior/middle frontal gyrus	L	−33	3	63	3.71	*
DLPFC/middle frontal gyrus	R	51	24	45	2.92	106
	R	48	33	33	2.82	*
DLPFC/middle frontal gyrus	R	21	57	30	2.67	65
DLPFC/precentral gyrus	R	36	3	60	3.10	519
DMPFC/superior frontal gyrus		0	−6	75	3.90	*
	L	−3	3	69	3.64	*
DMPFC/superior frontal gyrus	R	12	15	48	2.00	27
Middle temporal gyrus	L	−57	−48	6	2.88	82
Occipital lobe	R	15	81	12	3.39	537
	L	−21	−87	−12	2.81	*
Cerebellum	R	36	−66	−21	2.10	*
Cerebellum/fusiform gyrus	L	−33	−72	−18	3.07	*
Brainstem		0	−30	−12	2.53	74
**COMMON REGIONS OF DE-ACTIVATION (CONJUNCTION OF LABEL < OBSERVE AND REAPPRAISE < OBSERVE)**						
Amygdala/parahippocampal gyrus	L	−30	0	−21	2.98	134
Amygdala	R	24	−6	−15	2.04	10
VMPFC	L	−3	48	−3	2.55	60
	R	3	54	−9	2.26	*
Subgenual ACC/VMPFC	R	3	27	−3	3.12	94
Medial temporal/peri-amygdala	R	42	−3	−15	3.02	76
Fusiform gyrus	R	36	−42	−18	2.60	89
Parietal cortex/precuneus	L	−15	−42	48	3.07	64

*Conjunction analyses were thresholded with an uncorrected p-value of 0.003 combined with an extent threshold of 4 and 24 contiguous voxels for a priori amygdala and PFC ROIs, respectively, and 43 contiguous voxels for all other regions, correcting for multiple comparisons using AlphaSim*.

* *Denotes same cluster as immediately above. VLPFC, ventrolateral prefrontal cortex; DLPFC, dorsolateral prefrontal cortex; VMPFC, ventromedial prefrontal cortex; DMPFC, dorsomedial prefrontal cortex; ACC, anterior cingulate cortex*.

We also examined common *decreased* responses during Label and Reappraise relative to Observe (i.e., the conjunction of Label < Observe and Reappraise < Observe). As predicted, there was significantly less bilateral amygdala activation during Label and Reappraise relative to Observe (24, −6, −15; *t* = 2.04; 10 voxels; −30, 0, −21; *t* = 2.98; 134 voxels; see Figure 3), suggesting that both strategies were effective in emotion down-regulation at the neural level. Figure 5 shows extracted parameter estimates of bilateral amygdala activity during Label, Reappraise, and Observe relative to the non-emotional Shape Match condition (i.e., Label > Shape Match, Reappraise > Shape Match, and Observe > Shape Match) to illustrate that amygdala activity during affect labeling and reappraisal is significantly reduced relative to passive observation. As shown in Table 1 and Figure 3, other common decreased activations were seen in ventral medial PFC (VMPFC), subgenual anterior cingulate cortex (subACC), medial temporal lobe/peri-amygdala, fusiform gyrus, and a cluster spanning the parietal cortex and precuneus. Decreased VMPFC and subACC during affect labeling has been observed previously (Lieberman et al., 2007).

#### Differential responses during affect labeling and reappraisal

**Figure 4:**
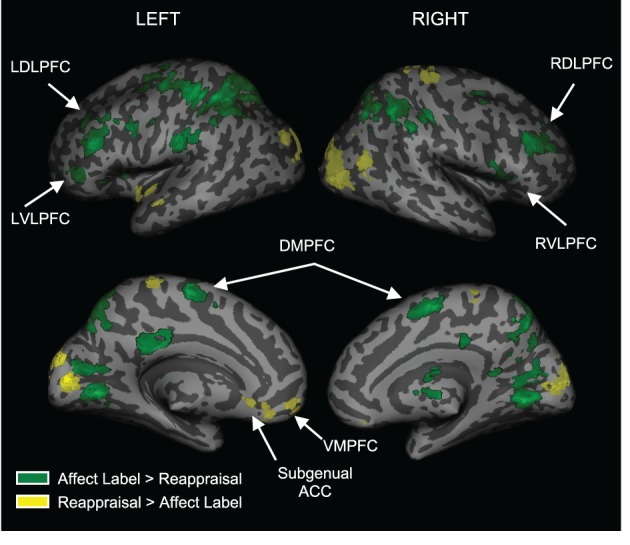
**Distinct activations for Labeling vs. Reappraisal**. Areas in green represent greater activation for Labeling relative to Reappraisal. Areas in yellow represent greater activation for Reappraisal relative to Labeling.

To examine regions differentially activated during affect labeling and reappraisal, we compared affect labeling and reappraisal directly (i.e., Label > Reappraise and Label < Reappraise). As seen in Table 2 and Figure 4, there was significantly greater activity during Label compared to Reappraisal in several emotion regulatory regions, including RVLPFC (54, 21, 6; *t* = 2.89; 48 voxels), LVLPFC (−45, 39, 3; *t* = 3.99; 42 voxels), RDLPFC (33, 33, 30; *t* = 4.32; 149 voxels; 27, 0, 57; *t* = 4.02; 25 voxels), LDLPFC (−36, 36, 27; *t* = 5.63; 93 voxels and −42, 0, 33; *t* = 3.88; 54 voxels), and posterior DMPFC/Supplementary motor area (0, 0, 57; *t* = 3.54; 95 voxels). After applying the Shape Match-based motor response mask, the activations in bilateral VLPFC and the more anterior portions of bilateral DLPFC (33, 33, 30 and −36, 36, 27) were no longer included. In other words, the greater activity seen in bilateral VLPFC and anterior DLPFC during Label > Reappraise does not appear to be a function of the basic motor response processes of affect labeling since these areas did not overlap with the activations associated with the non-emotional motor response task (i.e., Shape Match).

**Table 2:** **Differential activations for Labeling and Reappraising compared directly**.

		***x***	***y***	***z***	***t***	**Voxels**
**REGIONS OF ACTIVATION FOR LABEL > REAPPRAISE**						
VLPFC/inferior frontal gyrus	L	−45	39	3	3.99	42
VLPFC/inferior frontal gyrus	R	42	18	12	3.74	48
	R	54	21	6	2.89	*
DLPFC/middle frontal gyrus	L	−36	36	27	5.63	93
DLPFC/middle frontal gyrus	R	33	33	30	4.32	149
DLPFC/precentral gyrus^†^	L	−42	0	33	3.88	54
DLPFC/precentral gyrus^†^	R	27	0	57	4.02	25
DMPFC/supplementary motor area^†^		0	0	57	3.54	95
Posterior cingulate cortex	L	−3	−33	30	4.25	48
Precuneus^†^	R	12	−75	60	6.76	1720
Precentral gyrus^†^	L	−39	−15	66	4.65	*
Inferior parietal cortex^†^	L	−36	−45	48	6.00	*
	R	36	−48	42	4.97	*
Superior parietal cortex^†^	L	−27	−66	51	4.99	*
Interior parietal cortex^†^	L	−51	−24	21	4.42	76
Cerebellum^†^	L	9	−60	−15	6.76	1154
	L	−48	−57	−30	5.02	224
**REGIONS OF ACTIVATION FOR REAPPRAISE > LABEL**						
VMPFC		0	33	−21	4.23	28
VMPFC	L	−3	51	−9	4.30	29
Subgenual ACC		0	18	−12	3.60	40
Parietal cortex	R	15	−36	72	3.65	45
Occipital lobe	L	−15	−102	15	6.95	618
	R	15	−99	15	8.84	*

*Activations were thresholded using an uncorrected p-value of 0.005 combined with an extent threshold of 4 and 24 contiguous voxels for a priori amygdala and PFC ROIs, respectively, and 43 contiguous voxels for all other regions, correcting for multiple comparisons using AlphaSim*.

† *Signifies activations that overlapped with those seen during the basic motor response task (Shape Match)*.

* *Denotes same cluster as immediately above. VLPFC: ventrolateral prefrontal cortex. DLPFC: dorsolateral prefrontal cortex. VMPFC: ventromedial prefrontal cortex. DMPFC: dorsomedial prefrontal cortex. ACC: anterior cingulate cortex*.

**Figure 5:**
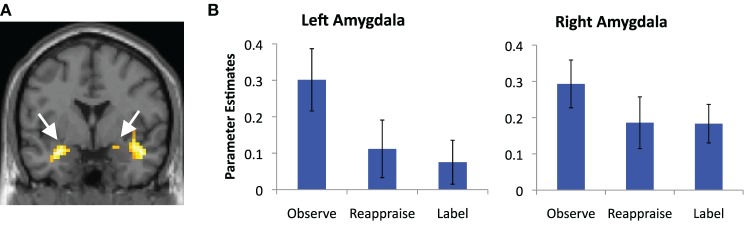
**Amygdala reductions. (A)** Magnetic resonance image showing common regions of bilateral amygdala de-activation during affect labeling and reappraisal relative to passive observation; and **(B)** parameter estimates of activity extracted from the portion of these clusters that lies within our anatomical amygdala ROIs (AAL) and plotted relative to the shape match control condition for illustration purposes. Error bars represent differences with respect to Shape Match.

There was no activity in any lateral PFC regulatory region that was greater during Reappraisal compared to Label. There was greater activity during Reappraisal compared to Label in VMPFC and subgenual ACC, which have been associated with emotion regulation in some studies. Also, of note, there were no significant differences in amygdala activation when comparing Reappraise and Label directly, suggesting that neither strategy was significantly more effective than the other in reducing amygdala activity.

## Discussion

Using fMRI, we examined the common and divergent patterns of neural activity associated with affect labeling and reappraisal of emotional experiences. Results are noteworthy in that, first, we found that affect labeling of one's own emotions produces a similar neural pattern as affect labeling of the emotional aspects of external stimuli in other samples, namely, increased RVLPFC and decreased amygdala (Lieberman et al., 2007).

Second, we observed that affect labeling and reappraisal were associated with significant overlapping activations in multiple prefrontal regions associated with regulatory processes, including bilateral VLPFC, bilateral DLPFC, and posterior DMPFC, as well as significant overlapping deactivations in bilateral amygdala. These findings are generally consistent with previous research examining each strategy separately (Ochsner and Gross, 2005; Berkman and Lieberman, 2009). More importantly, as the first well-powered study examining the two strategies in a single group of participants, these results also confirm that affect labeling and reappraisal share common neural mechanisms.

The extent of this overlap may seem surprising given that affect labeling and reappraisal are very different processes at an experiential level. Reappraisal involves conscious, deliberate, and effortful attempts to change cognitions and felt emotions. In contrast, affect labeling involves self-reflection and verbalization of one's current emotional state, and notably, does not involve any conscious attempts to change one's cognitions or emotions. One might begin to interpret the overlapping prefrontal activations observed in this study by examining possible sub-processes that are shared by these seemingly distinct emotion regulation strategies and subserved by the overlapping neural regions. Both reappraisal and affect labeling likely involve (1) long-term memory retrieval and selection processes to either facilitate the implementation of an alternate interpretation (reappraisal) or the identification of current feelings (labeling), (2) attention management and conflict resolution processes to deal with counterproductive or task-interfering cognitions, (3) working memory and self-reflection processes to facilitate self-monitoring for regulation success (reappraisal) or emotion identification (labeling), (4) verbal processing associated with self-talk (reappraisal) or emotion labeling, and of course, (5) inhibitory processing to dampen affective responding (Ochsner and Gross, 2005; Berkman and Lieberman, 2009; Kalisch, 2009). In some cases, reappraisal may even explicitly involve affect labeling (e.g., “I feel anxious looking at the spider but I am not in danger since it is just a photograph”). The regions commonly activated for reappraisal and labeling, particularly bilateral VLPFC and DLPFC, have been shown to play key roles in these processes (Fletcher and Henson, 2001; Ochsner and Gross, 2005; Berkman and Lieberman, 2009; Kalisch, 2009). However, given that these regions each subserve multiple types of processing and the present study was not designed to test for specific sub-processes, it is not possible to definitively conclude that the overlapping activations here represent specific common neurocognitive processes (Poldrack, 2006). Nevertheless, results do support a common neural basis for reappraisal and affect labeling. More generally, results provide support for the idea that intentional and incidental emotion regulation utilize similar neurocognitive mechanisms, despite distinct experiential qualities, explicit goals, and partially overlapping sub-processes.

Unexpectedly, results also indicated that affect labeling engaged many of the commonly activated prefrontal regions to a greater extent than reappraisal. The greater VLPFC and DLPFC activity seen during affect labeling should not be interpreted to mean greater emotion down-regulation as there were no significant differences in amygdala or other limbic activity between the two conditions, and it was the reappraisal condition that yielded larger reductions in self-reported unpleasantness. Additionally, given that these regions did not overlap with the activations during a non-emotional Shape Match motor response task, it also does not appear that these activations are due to the differences in motor processing between affect labeling and reappraisal. Nevertheless, it remains a possibility that the different response modalities of affect labeling and reappraisal may have influenced these results. For example, the greater PFC activation seen during affect labeling relative to reappraisal may reflect differential effort, task difficulty, attention, or engagement. For example, affect labeling involved selecting an option from three choices provided and pressing the appropriate button, a process involving potential evaluation by experimenters. In contrast, reappraisal involved more free-form cognitive processing with no physical response required or evaluation possible. As such, participants may have been more engaged in the affect labeling condition, knowing that their performance could be evaluated. Alternatively, it is possible that there was less variability in the processes engaged in the affect labeling condition across participants and this led to more consistent, and thus, significant activations. That is, in the affect labeling condition, participants thought about how they felt, examined the emotional response options, and chose one. While the felt emotions may have varied across participants, the process of emotion labeling was likely quite consistent. In contrast, the reappraisal condition likely involved many more diverse processes. Unfortunately, we do not have a metric to assess task adherence or specific cognitive processes utilized in each condition as a manipulation check.

Results also indicated that reappraisal was more effective than labeling in reducing self-reported feelings of unpleasantness, which is consistent with previous studies (Lieberman et al., 2011). However, despite these differences in self-reported unpleasantness, reappraisal and labeling yielded similar emotion down-regulation at the neural level, as indexed by similar amygdala deactivation. There are two explanations that may reconcile these seemingly disparate findings. First, it is possible that these findings reflect biases of demand characteristics. Specifically, for the reappraisal condition, participants were explicitly told the goal was to decrease their negative emotional responses, whereas the labeling condition did not involve such an instruction. Thus, given that people expect reappraisal to diminish emotional distress (Lieberman et al., 2011) self-reported distress reductions may be biased to some degree. On the other hand, amygdala activity is only one of several neural indicators of emotional responding and thus the effects may be due to differences in other undetected neurocognitive processes. It should also be noted that although the reductions in self-reported distress between affect labeling and reappraisal were statistically significant, they may not represent meaningful experiential differences.

Finally, results also indicated that while participants displayed a similar self-reported unpleasantness response pattern in general across conditions, as reflected by correlated ratings overall, ratings for labeling and reappraisal continued to be positively correlated when controlling for passive observation ratings. This suggests there may be consistency of emotion regulation efficacy across multiple regulation strategies.

The present study does have limitations. First, as mentioned above, this study did not include a measure of task adherence and therefore, it is possible that participants were not exclusively engaging in reappraisal and affect labeling when instructed to do so. Second, the affect labeling and reappraisal conditions in this study required different response modalities. Future studies would be improved by having participants simply label the emotion they are feeling internally to remove the behavioral and performance aspects unique to labeling in the present study. Third, we were not able to generate independent indices of amygdala reductions for labeling and reappraisal because both would be contrasted with the same amygdala activity during observe, and therefore correlations would be inflated and biased. Fourth, while personally-relevant affect labeling in this study yielded a similar pattern of neural activity as affect labeling of external stimuli in previous studies, there are likely to be some subtle differences (e.g., McRae et al., 2010). Future studies can address this issue by including both conditions in the same neuroimaging study. Also, our sample was unexpectedly disproportionately female and this limits the generalizability of our findings somewhat. Finally, the older age range of participants in the present study also limits the generalizability of the results to some extent. However, given the relatively strict inclusion and exclusion criteria, the present sample was quite healthy, minimizing the differences in neural functioning between this sample and younger adults that would stem from illness or disease more commonly seen in older adults in general. Furthermore, we note that most studies of emotion regulation have been conducted in younger adult samples (often college students), and thus, far less is known about neural emotion regulation in older adults (e.g., St. Jacques et al., 2010). Therefore, our focus on a sample of healthy older adults (aged 55–75) makes an important contribution by showing that there is stability in neural emotion regulation patterns later in life (cf. Ochsner et al., 2002; Lieberman et al., 2007).

In conclusion, results suggest common neurocognitive mechanisms supporting affect labeling and reappraisal, thereby providing support for the idea that intentional and incidental emotion regulation involve overlapping neural mechanisms. In addition to providing insights into this pervasive and influential component of human experience, results also have possible clinical implications. For example, results suggest that an individual who is able to effectively utilize one strategy may be proportionately able to utilize another strategy. Accordingly, strategies to strengthen or otherwise increase the effectiveness of one strategy may have far reaching effects, acting to incidentally increase the effectiveness of other regulation strategies. For example, ongoing work in our lab is exploring the idea of affect labeling as a relatively simple means to improve emotion regulation ability. Conversely, dysfunctions in these regulatory regions would unfortunately extend to multiple emotion regulation processes, suggesting a possible reason why some emotional disorders, such as depression can be so difficult to treat. Future research should further examine the specific neurocognitive processes that underlie intentional and incidental emotion regulation, as well as examine possible means for enhancing the effectiveness of these strategies.

### Conflict of interest statement

The authors declare that the research was conducted in the absence of any commercial or financial relationships that could be construed as a potential conflict of interest.

## Footnotes

^1^A major goal of the larger intervention study was to examine the effects of mindfulness meditation training on neural, psychological, and immune system functioning in healthy older adults. Thus, our inclusion and exclusion criteria were fairly strict in order to obtain a sample of physically and mentally healthy older adults naïve to mind-body practice and free of medications and/or conditions that might artificially influence immune systems functioning.

^2^The specific IAPS images included in this study were as follows. Observe: 3530, 9250, 9480, 9253, 1111, 3102, 6250, 1070, 6836, 9041, 6540, 5940, 3261, 3350, 1112, 3000, 8475, 9570, 6242, 2141. Label: 3068, 1201, 1726, 6571, 2710, 8485, 3140, 1301, 6831, 3230, 3030, 9921, 1114, 3280, 2750, 6260, 3064, 6555, 2800, 1030. Reappraise: 3000, 6313, 1113, 9430, 2590, 6821, 1090, 9230, 2661, 3051, 6510, 9050, 3062, 1080, 2053, 3010, 1200, 3180, 2692, 2900.1.
